# Risk of metabolic syndrome among children living in metropolitan Kuala Lumpur: A case control study

**DOI:** 10.1186/1471-2458-11-333

**Published:** 2011-05-18

**Authors:** Bee S Wee, Bee K Poh, Awang Bulgiba, Mohd N Ismail, Abdul T Ruzita, Andrew P Hills

**Affiliations:** 1 Department of Nutrition and Dietetics, Faculty of Allied Health Sciences, Universiti Kebangsaan Malaysia, 50300 Kuala Lumpur, Malaysia; 2 Faculty of Medicine and Health Sciences, Universiti Sultan Zainal Abidin, 20400 Kuala Terengganu, Malaysia; 3 Julius Centre University of Malaya, Faculty of Medicine, 50603 Kuala Lumpur, Malaysia; 4 Institute of Health and Biomedical Innovation, Queensland University of Technology, Brisbane, Australia

## Abstract

**Background:**

With the increasing prevalence of childhood obesity, the metabolic syndrome has been studied among children in many countries but not in Malaysia. Hence, this study aimed to compare metabolic risk factors between overweight/obese and normal weight children and to determine the influence of gender and ethnicity on the metabolic syndrome among school children aged 9-12 years in Kuala Lumpur and its metropolitan suburbs.

**Methods:**

A case control study was conducted among 402 children, comprising 193 normal-weight and 209 overweight/obese. Weight, height, waist circumference (WC) and body composition were measured, and WHO (2007) growth reference was used to categorise children into the two weight groups. Blood pressure (BP) was taken, and blood was drawn after an overnight fast to determine fasting blood glucose (FBG) and full lipid profile, including triglycerides (TG), high-density lipoprotein cholesterol (HDL-C), low-density lipoprotein cholesterol (LDL-C) and total cholesterol (TC). International Diabetes Federation (2007) criteria for children were used to identify metabolic syndrome.

**Results:**

Participants comprised 60.9% (n = 245) Malay, 30.9% (n = 124) Chinese and 8.2% (n = 33) Indian. Overweight/obese children showed significantly poorer biochemical profile, higher body fat percentage and anthropometric characteristics compared to the normal-weight group. Among the metabolic risk factors, WC ≥90^th ^percentile was found to have the highest odds (OR = 189.0; 95%CI 70.8, 504.8), followed by HDL-C≤1.03 mmol/L (OR = 5.0; 95%CI 2.4, 11.1) and high BP (OR = 4.2; 95%CI 1.3, 18.7). Metabolic syndrome was found in 5.3% of the overweight/obese children but none of the normal-weight children (*p *< 0.01). Overweight/obese children had higher odds (OR = 16.3; 95%CI 2.2, 461.1) of developing the metabolic syndrome compared to normal-weight children. Binary logistic regression showed no significant association between age, gender and family history of communicable diseases with the metabolic syndrome. However, for ethnicity, Indians were found to have higher odds (OR = 5.5; 95%CI 1.5, 20.5) compared to Malays, with Chinese children (OR = 0.3; 95%CI 0.0, 2.7) having the lowest odds.

**Conclusions:**

We conclude that being overweight or obese poses a greater risk of developing the metabolic syndrome among children. Indian ethnicity is at higher risk compared to their counterparts of the same age. Hence, primary intervention strategies are required to prevent this problem from escalating.

## Background

Metabolic syndrome is recognised as the clustering of risk factors of obesity, insulin resistance, dyslipidemia and hypertension associated with the subsequent development of cardiovascular disease and type 2 diabetes [1]. With the increasing prevalence of overweight and obesity worldwide, especially in children and youth, the "paediatric metabolic syndrome" has received increasing attention from a public health perspective [2].

In Asia, there is a trend towards an increase in the prevalence of the metabolic syndrome and cardiovascular disease [3-5]. Abdominal obesity is arguably the key factor underlying the development of insulin resistance and the metabolic syndrome [6]. However, higher body fat, truncal subcutaneous fat, intra-abdominal fat and ectopic fat deposition (liver and fat) worsens the situation and is particularly evident in South Asian/Asian Indian adults and children [7].

The mechanisms underlying the ethnic differences in metabolic risk are not fully understood. However both genetic and environmental factors are likely to be important in determining differential levels of central obesity and metabolic disturbances [8]. Ethnic differences have also been considered as a major factor in expressing the syndrome. For example, Lee et al. in a total of 22,952 participants from Australia, Japan, Korea and Samoa, reported that the prevalence of the metabolic syndrome was lowest among the Japanese and highest among the Samoans [9]. In another study, Pollestad-Kolsgaard revealed that the metabolic syndrome was more frequent among children and adolescents from Middle Eastern and South Asian origins (Pakistani, Tamil and Turkish) than Norwegians [10].

Malaysia is acknowledged for its ethnic diversity with the population comprising three main ethnic groups: Malays, Chinese and Indians. Kuala Lumpur, the capital city and its suburbs form a metropolitan area that includes these ethnic groups who live in the same environment but practice somewhat different cultures and lifestyles. Ethnic and gender influences among children with the metabolic syndrome have not been reported to date in Malaysia. Therefore, the aim of this study was to compare the metabolic syndrome risk factors among overweight/obese and normal-weight children as well as its association with ethnicity and gender.

## Methods

### Study Design

This was a case control study conducted on primary school children aged nine to twelve years in Kuala Lumpur and its metropolitan suburbs between October 2007 and November 2008. Nine schools representative of all socio-economic groups were randomly selected from a total of 80 schools in the respective areas. Approval was obtained from the Medical Research and Ethics Committee of the Universiti Kebangsaan Malaysia, Ministry of Education and school principals before the research commenced.

### Screening of participants and selection criteria

In order to determine the prevalence of overweight/obese among the children, screening was undertaken in all classes in the selected schools by measuring height and weight and calculating the Body Mass Index (BMI). The WHO BMI-for-age growth reference [11] was used to classify participants as case or control based on their decimal age which was determined using date of birth and date of measurement.

Inclusion criteria were: (i) aged between 9-12 years; (ii) overweight/obesity was categorised as BMI greater than +1SD from the mean or normal-weight which was categorised as BMI less than or equal to median; (iii) healthy without any disease and not on any medication at the time of the study. Children were excluded if they had any disease or syndrome that may affect the fat distribution or body composition, such as Cushing syndrome or hypothyroidism, based on information provided by the parents; writing or speaking problems; and not fasted on the day of data collection.

**Figure 1:**
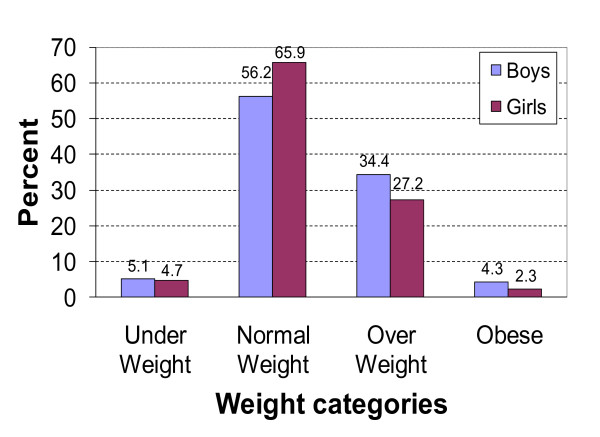
**Distribution of weight status according to WHO 2007**.

A total of 2770 children were screened from the nine schools and categorised as follows: underweight: 4.9%; normal-weight: 60.9%; overweight: 30.9% and obese: 3.3%. Accordingly, the prevalence of overweight/obesity was 34.2% (see Figure 1).

### Sampling Method

Based on the prevalence of overweight/obesity of 34.2% in the population, the sample size was determined using the EPI INFO version 6 software. As the study was a comparison study between normal-weight *vs*. overweight/obese, a case control study with power of study and confidence level set at 80% and 95% respectively, with the odds ratio estimated as 2.0; based on a study by Garnett et al., where overweight/obese children had odds of 7 times of developing cardiovascular disease clustering compared to normal-weight children [12]. Thus, the minimum sample size was 148. The final sample size determined with provision for a 30% drop-out rate was 192 for each of the normal-weight and overweight/obese groups.

### Data collection

Data collection was carried out between May and November 2008 on participants who matched inclusion criteria, and had written parental informed consent. Two post graduate nutrition students who had undergone training on anthropometric assessments examined the subjects.

### Socio-demographic information

Each child was given a copy of the socio-demographic form which was filled in by the parents. Information on birth weight, household incomes, number of children in the family as well as family history of diseases that were related to the metabolic syndrome such as hypertension, diabetes mellitus, heart diseases or hypercholesterolemia or the combination of them were obtained.

### Anthropometric measurements

#### i) Body weight and height

Weight was recorded in light clothing to the nearest 0.1 kg on a SECA Model 813 digital weighing scale (SECA, Germany) and height was measured without shoes to the nearest 0.1 cm using a SECA Stadiometer Model 214 (SECA, Germany). The average of the two values for each measurement was used in the data analysis. Body mass index (BMI) was calculated from weight and height.

#### ii) Waist circumference

Waist circumference (WC) was measured using a SECA Model 200 tape (SECA, Germany) to the nearest 0.1 cm over skin, midway between the tenth rib and the iliac crest. According to the protocol used by Sung et al. [13], waist circumference >90^th ^percentile was considered as a risk for the development of the metabolic syndrome [14].

### Body composition measurement

Fat mass (FM) and fat-free mass (FFM) were estimated using body fat analyzer, Maltron BioScan 916 (Maltron, UK). Participants fasted overnight prior to the day of data collection and measurement was taken after the bladder was emptied and the participant had rested in a supine position for 5-10 minutes.

### Blood pressure measurement

Arterial blood pressure was measured manually using a mercury sphygmomanometer with a suitable cuff size for each participant after a 5-min rest in the supine position. Systolic blood pressure was determined by the onset of the tapping Korotkoff sound while diastolic was determined after the disappearance of the Korotkoff sound. Systolic blood pressure ≥130 mmHg or diastolic ≥85 mmHg were used as cut-off points for metabolic syndrome [14].

### Biochemical measurements

A venous blood sample was collected after a 10-h fast using the standard venepuncture technique by a trained phlebotomist. A full lipid profile consisted of total cholesterol (TC), triglycerides (TG), high-density lipoprotein cholesterol (HDL-C), low-density lipoprotein cholesterol (LDL-C), and fasting blood glucose (FBG). FBG was measured by hexokinase method, TC and TG were measured by enzymatic reaction while HDL cholesterol was determined by catalase method. LDL cholesterol was calculated by using Friedewald formula. All biochemical analyses were undertaken by a private laboratory with international quality control Certificate of Accreditation (ISO 15189).

### Diagnostic criteria

Metabolic syndrome was diagnosed based on the International Diabetes Federation's paediatric definition [14], namely waist circumference ≥ 90^th ^percentile plus two or more of the following indices for all boys and girls:

a. Triglycerides ≥150 mg/dL (1.7 mmol/L)

b. Blood pressure (Systolic ≥130 mmHg or Diastolic ≥85 mmHg)

c. Fasting blood glucose ≥100 mg/dL (5.6 mmol/L)

d. High-density lipoprotein cholesterol ≤40 mg/dL (1.03 mmol/L)

### Statistical Analysis

All data was entered and analysed using Statistical Package for Social Sciences version 15.0 (SPSS, Inc., Chicago, IL, USA). Appropriate statistical analysis was carried out with a two-sided alpha level of 0.05 or 95% confidence interval. For continuous data, descriptive analyses used means and standard deviation (SD). Independent t-test as well as Mann-Whitney U test (where assumptions for the t test could not be met) was used for comparison of physical and biochemical profile. Fisher's Exact, Pearson's chi-square test and odds ratio was performed to look for relationships between categorical variables. Binary logistic regression analysis was performed to examine the relationship between socio-demographic characteristics: age, gender, ethnicity as well as family history of diseases with the metabolic syndrome and obesity. Observed associations were expressed as OR with 95% confidence intervals.

## Results

Among the screened participants, 896 matched the inclusion criteria; however only 447 agreed to participate in the study. Forty-five participants withdrew on the day of data collection because they were either unwell, afraid to have their blood drawn, or were not fasted for at least 10 hours; hence a final sample of 402 was achieved.

Out of the 402 children, 193 were boys and 209 were girls with 60.9% (n = 245) being Malays, 30.9% (n = 124) Chinese and 8.2% (n = 33) Indians. Overweight/obese children came from families with higher income (mean USD1199 ± 981) compared to normal-weight children (mean USD870 ± 767). Although overweight/obese children had a lower mean birth weight (3.09 kg ± 0.49) than normal-weight children (3.11 kg ± 0.56), they were not significantly different. Similar results were also found for numbers of children in the family and for age. Socio-demographic characteristics are shown in Table 1.

**Table 1:** Socio-demographic characteristics of the sample [(mean ± SD; n (%)]

Description	Overweight/obese (N = 209)	Normal-weight (N = 193)	t test *P *value
Age (years)	11.0 ± 0.83	11.0 ± 0.9	0.769
Gender, N (%)			
Boys	102 (48.8)	92 (47.7)	
Girls	107 (51.2)	101 (52.3)	N/A
Ethnicity, N (%)			
Malays	128 (61.2)	117 (60.6)	
Chinese	61 (29.1)	63 (32.6)	N/A
Indian	20 (9.7)	13 (6.8)	
Household income (USD)	1199 ± 981	870 ± 767	0.112
Birth weight (kg)	3.09 ± 0.49	3.11 ± 0.56	0.774
Number of children in the family	2.3 ± 1.8	2.2 ± 1.3	0.869

Note: 1 MYR = 0.322217 USD

N/A: not applicable

Anthropometric and biochemical characteristics of the children are shown in Table 2. Overweight/obese children had significantly worse clinical profiles and higher anthropometric parameters [height, weight, BMI, WC, hip circumference (HC), fat mass (FM) (%), waist hip ratio (WHR), waist-to-height ratio (WHtR), TG, HDL-C, SBP and DBP, *p *< 0.001; LDL-C *p *< 0.05] compared to normal-weight children except for TC and FBG.

**Table 2:** Comparison of anthropometric and biochemical characteristics of groups (mean, 95%CI)

Description	Overweight/obese	Normal-weight
	(N = 209)	(N = 193)
	Mean (95%CI)	Mean (95%CI)
Height (cm) †	146.7 (145.7, 147.8) **	139.7 (138.6, 140.9)
Weight (kg) †	54.4 (52.9, 56.0) **	31.2 (30.3, 32.0)
BMI (kg/m^2^) †	25.1 (24.6, 25.6) **	15.8 (15.5, 16.1)
Fat mass (%)†	29.8 (28.2, 31.4) **	18.6 (17.5, 19.6)
Fat free mass (%)†	70.2 (68.6, 71.8) **	81.4 (80.4, 82.5)
Waist circumference (cm) †	77.2 (76.0, 78.5) **	55.9 (55.1, 56.7)
Hip circumference (cm) †	91.5 (90.4, 92.6) **	72.3 (71.4, 73.1)
Waist height ratio†	0.5 (0.5, 0.5) **	0.4 (0.3, 0.4)
Waist hip ratio†	0.8 (0.8, 0.9) **	0.7 (0.7,0.8)
Fasting blood glucose (mmol/L) †	5.0 (4.9, 5.1)	5.0 (4.9, 5.0)
Triglycerides (mmol/L) #	1.1 (1.0, 1.2)**	0.8 (0.7, 0.9)
HDL-cholesterol (mmol/L) †	1.3 (1.2, 1.3)**	1.5 (1.4, 1.5)
LDL-cholesterol (mmol/L) #	3.0 (2.9, 3.1)*	2.9 (2.8, 3.0)
Total cholesterol (mmol/L)#	4.8 (4.6, 4.9)	4.7 (4.6, 4.8)
SBP (mmHg) †	109.4 (108.0, 110.7)**	100.9 (99.7, 102.2)
DBP(mmHg) †	67.1 (65.5, 68.5)**	64.4 (63.0, 65.7)

†denotes independent t test

#denotes Mann-Whitney U test

** significant at *p *< 0.001

*significant at *p *< 0.05

The distribution of five risk factors of the metabolic syndrome and the full syndrome with its odds ratio among the children are shown in Table 3. More than 80% of the overweight/obese children had a WC ≥90^th ^percentile (*p *< 0.001) compared to 2.6% of the normal-weight children. Low HDL-C was present in 19.7% of the overweight/obese *vs*. 4.7% of the normal-weight children (*p *< 0.001). Similarly for triglycerides (12.5% *vs*. 4.2%) (*p *< 0.01) and high blood pressure (6.3% *vs*. 1.6%) (*p *< 0.05). Metabolic syndrome was diagnosed in 5.3% of the overweight/obese children and none of the normal-weight children. Among the risk factors, a high WC gave the highest odds of 189.0 (95%CI 70.8, 504.8) of developing metabolic syndrome followed by low HDL-C (OR = 5.0; 95%CI 2.4, 11.1), high blood pressure (OR = 4.2; 95%CI 1.3, 18.7); high triglycerides (OR = 2.5; 95%CI 0.7, 9.3), and high FBG (OR = 1.5; 95%CI 0.4, 5.8). Overweight/obese children were found to have 16.3 the odds (95%CI 2.2, 461.1) of developing the metabolic syndrome compared to their normal-weight counterparts.

**Table 3:** Comparison of metabolic components between overweight/obese and normal-weight children

Parameter	Overweight/obese (N = 209) n (%)	Normal-weight (N = 193) n (%)	**OR (95%CI)**^**a**^
WC ≥90^th ^Percentile#			
Yes	176 (84.2)^d^	5 (2.6)	189.0 (70.8, 504.8)
No	33 (15.8)	188 (97.4)	1.0
FBG ≥5.6 mmol/L#			
Yes	12 (5.8)	10 (5.2)	1.5 (0.4, 5.8)
No	196 (94.2)	182 (94.8)	1.0
TG ≥1.7 mmol/L#			
Yes	26 (12.5)^c^	8 (4.2)	2.5 (0.7, 9.3)
No	182 (87.5)	184 (95.8)	1.0
HDL-C ≤1.03 mmol/L#			
Yes	41 (19.7) ^d^	9 (4.7)	5.0 (2.4, 11.1)
No	167 (80.3)	183 (95.3)	1.0
High BP†			
Yes	13 (6.3)^b^	3 (1.6)	4.2 (1.3, 18.7)
No	195 (93.8)	190 (98.4)	1.0
Metabolic Syndrome†			
Yes	11 (5.3) ^c^	0 (0)	16.3 (2.2, 461.1)^e^
No	198 (94.7)	193 (100)	1.0

†Denotes Fisher's Exact Test

# Denotes Pearson's Chi-square Test

^b ^significant at *p *< .05; ^c ^significant at *p *< .01; ^d ^significant at *p *< .001

^a ^OR calculated from logistic regression test by using enter method

^e ^OR was calculated by adding 0.5 into the cell with 0 value

Table 4 illustrates results for the clustering of risk factors. Only 12% of the overweight/obese children were free of any risk factors compared to 83.9% of the normal-weight children. More than 50% of the overweight/obese children had 1 risk factor *vs*. only 14.0% of the normal-weight children. As for two risk factors, 28.2% of overweight/obese children had these *vs*. 2.1% of normal-weight children. None of the normal-weight children had three or four risk factors compared to 3.8% and 1.4% of the overweight/obese children, respectively. Overweight/obese children had odds of 27.4 (95%CI 15.1, 49.6) of developing the metabolic syndrome compared to normal-weight children with one risk factor. The odds increased to 95.6 (95%CI 31.9, 286.2) for those with two risk factors.

**Table 4:** Clustering of metabolic risk factors in obese and normal-weight children

Metabolic Syndrome Risk Components	Overweight/obese (N = 209) n (%)	Normal-weight (N = 193) n (%)	**OR (95%CI)**^**a**^
No risk factor	25 (12.0)	162 (83.9)	1.0
1 risk factor	114 (54.5)	27 (14.0)	27.4 (15.1, 49.6)**
2 risk factors	59 (28.2)	4 (2.1)	95.6 (31.9, 286.2)**
3 risk factors	8 (3.8)	0 (0)^b^	0 (0.0, 0.1)
4 risk factors	3 (1.4)	0 (0) ^b^	0 (0.0, 0.2)

^a ^OR calculated from logistic regression test by using enter method

^b ^OR was calculated by adding 0.5 into the cell with 0 value

To determine the influence of family history of disease, age, gender and ethnicity with the risk of developing the metabolic syndrome, a univariate binary logistic regression test was conducted, the results of which are shown in Table 5. Children with a family history of diseases had an odds of 3.4 (95%CI 0.8, 16.9) of developing the metabolic syndrome compared to children without a family history. However, there was no significant association between family history of disease with the metabolic syndrome group.

**Table 5:** Metabolic syndrome and associated factors

Variables	Metabolic syndrome (N = 11) n (%)	No Metabolic syndrome (N = 391) n (%)	OR (95%CI) †
With family history			
Yes	6 (4.3)	132 (95.7)	3.4 (0.8, 16.9)
No	3 (1.3)	226 (98.7)	1.0
Age			
9-10	3 (1.5)	198 (98.5)	1.0
11-12	8 (4.0)	191 (96.0)	2.8 (0.7, 10.6)
Gender			
Boys	3 (1.6)	190 (98.4)	1.0
Girls	8 (3.9)	199 (96.1)	2.5 (0.7, 9.7)
Ethnicity#			
Malay	6 (2.5)	238 (97.5)	1.0
Chinese	1 (0.8)	122 (99.2)	0.3 (0.0, 2.7)
Indian	4 (12.1)	29 (87.9)	5.5 (1.5, 20.5)

**†**Univariate logistic regression test

# Other races was excluded from the analysis because the percentage was too low (0.2%)

Children in the older age group (11-12 years) had odds of 2.8 (95%CI 0.7, 10.6) of developing the metabolic syndrome compared to those in the younger age group (9-10 years). Furthermore, girls had odds of 2.5 (95%CI 0.7, 9.7) of developing the metabolic syndrome compared to boys. Overall, Indians had significantly higher odds (OR = 5.5; 95%CI 1.5, 20.5) of developing metabolic syndrome compared to Malay children; with the Chinese having the lowest odds (OR = 0.3; 95%CI 0.0, 2.7) compared to Malays.

## Discussion

The prevalence of overweight and obese children in our study in metropolitan Kuala Lumpur was 34.2%; higher than in an earlier study by Ismail et al. who reported an increase from 20.7% in 2002 to 26.5% in 2008 using the WHO growth reference in 6-12 year-old children in Peninsular Malaysia [15]. With the increasing trend of childhood obesity worldwide, it is not surprising that the metabolic syndrome is present in 5.3% of our overweight/obese population aged 9-12 years. This result is comparable to a US study (4.2%) in children and adolescents aged 12-19 years in the NHANES III survey [16], another US study in Kansas in 7-9 year-old children (5%) [17], and in Denmark (5.4%) [18]. In Kuala Lumpur, another study reported that metabolic syndrome was found in 1.3% of children aged 7 to 9 years [19].

Our results revealed that with the exception of FBG and TC, overweight/obese children had significantly higher anthropometric and biochemical indices compared to normal-weight counterparts. Misra and Vikram suggest that high FBG will only be visible when other metabolic components start appearing [20]. Thus, it may take many years for FBG to be visibly high among many overweight/obese children. Moreover, the IDF criteria for diagnosis of the metabolic syndrome for children use the same cut-offs as for adults except WC [14]. Hence, the level of the other metabolic components may not be severe enough for them to be diagnosed with the metabolic syndrome.

Being overweight/obese poses a higher risk of developing the metabolic syndrome with odds of 16.3 as compared to normal-weight individuals. WC is typically used as a surrogate measure of abdominal obesity. With the attendant health risks of central fat distribution it is of little surprise that WC is the first criteria in determining the risk of the metabolic syndrome. However, there are no age-, gender- and ethnic-specific WC standards for the Asian childhood population, with the exception of the values based on a database of Hong Kong children [13]. Accordingly, it would be extremely useful to develop a WC cut-off points for each country so that meaningful comparisons could be made.

The presence of more risk factors also predisposed overweight/obese children to the metabolic syndrome. A Hungarian study reported that 76.7% of the obese children assessed had either one, two or three risk factors [21] which is comparable with the 86.5% of overweight/obese children in our study with one, two or three risk factors. Individuals with one risk factor in our study have 27.4 the odds of developing the metabolic syndrome, and even higher among those with two risk factors (OR = 95.6).

Our data revealed that Indians have higher odds (OR = 5.5) of developing the metabolic syndrome compared to Malays, with Chinese having the lowest risk. Ethnic differences are widely reported, for example Cook et al. showed that the prevalence of the metabolic syndrome was higher in whites (4.8%) and Mexican Americans (5.6%) compared to African American children (2.0%) [16]. Similarly, Weiss et al. found that white children were at greater risk of developing the metabolic syndrome than African American children [22]. In Singapore, Malay women were found to be more prone to developing hypertension in association with insulin resistance compared to Chinese and Indians [23].

We found no significant association between age and gender with the metabolic syndrome. However, other studies have presented opposing viewpoints, for example 32.2% of girls compared to 40% of boys aged 5-18 years in Latin America had the metabolic syndrome [24]. Similarly, Agirbasli et al. reported more Turkish boys (76.7%) having the metabolic syndrome compared to girls [25]. This is in contrast to a study conducted by Ferreira et al. who used NCEP ATP III diagnostic criteria and classified 10.7% of boys and 25% of girls with the metabolic syndrome [26]. Some have suggested that higher numbers of girls are reported to have the metabolic syndrome due to hormonal changes and subsequent central body fat accumulation, especially during puberty [27].

For children with a family history of non-communicable diseases, no significant association was found with the metabolic syndrome. Nevertheless, Pankow et al., revealed that children who had at least one parent with the syndrome (insulin resistance) had statistically lower insulin sensitivity expressed as M_Ibm_, and higher fasting insulin after adjustment for gender, ethnic, age and Tanner stage [28]. Mean BMI, WC, WHR, triceps and subscapular skinfolds and percentage body fat were also significantly higher in children of affected parents.

The major limitation of our study was the lack of association between age, gender and ethnicity with the metabolic syndrome which may be due to the small sample size with possibility of type 2 error, given the low response rate. However, we believe that there may be still be a link between some factors and the outcome despite the non-significance of the odds ratios. There is a real possibility that the non-significance is merely due to the small sample size rather than true non-significance. Another limitation was there are some odds ratio and 95%CI which are very high. The wide 95%CI is caused by the small sample size (resulting in high standard error) and wide disparity between the 2 groups. However, the OR remains significant and there were no outliers which could influence the findings of the study.

In summary, this study is the first undertaken in Kuala Lumpur and its metropolitan suburb to assess the metabolic syndrome and its influence of gender and ethnicity in upper primary school children. Even at the young age of 9-12 years we detected that 5.3% of overweight/obese participants had the metabolic syndrome. Urgent action is needed to halt the progression of risk factors and to safeguard the future health of Malaysian children and adolescents.

## Conclusions

The present findings reveal that 5.3% of overweight/obese children in this study have the metabolic syndrome with only 12% of the overweight/obese group being free from the metabolic syndrome risk factors. This is in sharp contrast to 83.9% of the normal-weight children who were free from all risk factors. In addition, children of Indian ethnicity had the highest risk, followed by Malays and Chinese. The findings from this study provide a rationale for public health specialists to institute primary intervention strategies to increase awareness and promote healthy lifestyles in schools thus preventing the problem from becoming more widespread.

## Competing interests

The authors declare that they have no competing interests.

## Authors' contributions

BSW: study design, development of research protocol, data collection, data analysis, interpretation of data, and drafting of the manuscript. BKP: chief investigator of MOSTI grant, development of research protocol, supervision of data collection, interpretation of results, and editing of manuscript. AB: study design, data analysis and interpretation, editing of manuscript. MNI: chief investigator of IAEA project, development of research protocol, and revision of manuscript. ATR: development of research protocol, revision of the manuscript. APH: collaborative investigator on IAEA project, development of research protocol, revision of manuscript. All authors read and approved the final manuscript.

## Pre-publication history

The pre-publication history for this paper can be accessed here:

http://www.biomedcentral.com/1471-2458/11/333/prepub
